# Pressure overload-induced systolic heart failure is associated with characteristic myocardial microRNA expression signature and post-transcriptional gene regulation in male rats

**DOI:** 10.1038/s41598-023-43171-1

**Published:** 2023-09-26

**Authors:** Mihály Ruppert, Sevil Korkmaz-Icöz, Bettina Benczik, Bence Ágg, Dávid Nagy, Tímea Bálint, Alex Ali Sayour, Attila Oláh, Bálint András Barta, Kálmán Benke, Péter Ferdinandy, Matthias Karck, Béla Merkely, Tamás Radovits, Gábor Szabó

**Affiliations:** 1 Experimental Research Laboratory, Heart and Vascular Center, Semmelweis University, Városmajor u. 68, 1122 Budapest, Hungary; 2 Department of Cardiac Surgery, University of Heidelberg, Heidelberg, Germany; 3 Department of Cardiac Surgery, University Hospital Halle (Saale), Halle, Germany; 4 Pharmahungary Group, Szeged, Hungary; 5 Cardiometabolic and HUN-REN–SU System Pharmacology Research Group, Department of Pharmacology and Pharmacotherapy, Semmelweis University, Budapest, Hungary

**Keywords:** Molecular medicine, Molecular biology, Non-coding RNAs, Transcriptomics

## Abstract

Although systolic function characteristically shows gradual impairment in pressure overload (PO)-evoked left ventricular (LV) hypertrophy (LVH), rapid progression to congestive heart failure (HF) occurs in distinct cases. The molecular mechanisms for the differences in maladaptation are unknown. Here, we examined microRNA (miRNA) expression and miRNA-driven posttranscriptional gene regulation in the two forms of PO-induced LVH (with/without systolic HF). PO was induced by aortic banding (AB) in male Sprague–Dawley rats. Sham-operated animals were controls. The majority of AB animals demonstrated concentric LVH and slightly decreased systolic function (termed as AB_LVH_). In contrast, in some AB rats severely reduced ejection fraction, LV dilatation and increased lung weight-to-tibial length ratio was noted (referred to as AB_HF_). Global LV miRNA sequencing revealed fifty differentially regulated miRNAs in AB_HF_ compared to AB_LVH_. Network theoretical miRNA-target analysis predicted more than three thousand genes with miRNA-driven dysregulation between the two groups. Seventeen genes with high node strength value were selected for target validation, of which five (*Fmr1*, *Zfpm2*, *Wasl*, *Ets1, Atg16l1*) showed decreased mRNA expression in AB_HF_ by PCR. PO-evoked systolic HF is associated with unique miRNA alterations, which negatively regulate the mRNA expression of *Fmr1*, *Zfmp2*, *Wasl*, *Ets1* and *Atg16l1*.

## Introduction

Sustained pressure overload (PO) of the left ventricle (LV) (e.g., aortic stenosis [AS], aortic coarctation or arterial hypertension) induces the development of pathological LV myocardial hypertrophy (LVH). PO-evoked LVH is typically characterized by LV wall thickening, impaired diastolic function and preserved, or mildly reduced, systolic function^1^. These characteristic features develop relatively soon after PO is established, and in some patients and experimental animals none or only a slight and slow further progression could be observed^2^. On the other hand, rapid transition from pathological LVH into congestive systolic heart failure (HF) might take place^3^.

Importantly, the severity of PO has been identified as a strong predictor for the development of systolic HF by prior experimental studies. Accordingly, in small animal models of aortic banding (AB), the more severe constriction of the aorta (leading to a greater degree of PO) was found to be associated with a higher risk for LV dilatation and contractile dysfunction^4^. Nevertheless, it is also worth mentioning that even under laboratory conditions (where the severity of PO is standardized and the potential confounding effects of different comorbidities do not prevail) only a subgroup of experimental animals manifest dilated cardiomyopathy with decompensated systolic function^3,5^.

Data are scarce regarding the molecular mechanisms that drive the differences in maladaptation of the myocardium in response to chronic PO. Over the last decades, the parallel improvement of omics technologies and bioinformatics has made it possible to approach this scientific question in an unbiased manner^6^. Among the different omics methods, myocardial microRNA (miRNA) transcriptomics in particular has gained lot of attention^7^. miRNA are short, non-coding RNA sequences that regulate gene expression on the posttranscriptional level. Of great interest, screening of myocardial miRNA expression followed by miRNA-based target gene prediction has been found to be a useful tool for the identification of novel molecular targets in different cardiovascular pathologies^8–12^.

It also worth mentioning that the dysregulation of numerous miRNAs has been already reported in PO-induced pathological LVH^13,14^. Nonetheless, whether the two forms of PO-evoked LVH (the more progressive form with LV dilatation and decompensated systolic function versus the classical form with concentric LVH and preserved systolic function, respectively) show characteristic differences on the miRNA level leading to distinct gene expressional changes has not yet been investigated. Importantly, this type of comparison might help to filter out those maladaptive alterations of PO-evoked molecular changes which might be responsible for the deterioration of systolic function and the dilation of the LV.

Upon this, in the current study we aimed to investigate LV miRNA expressional changes and miRNA-driven posttranscriptional gene regulations in AB rats with and without systolic HF in order to identify novel molecular targets of systolic HF.

## Results

### Model characterization

Invasive hemodynamic measurement indicated markedly increased arterial blood pressure in the AB_LVH_ (systolic blood pressure [SBP]: 218 ± 8 vs. 146 ± 6 mmHg, diastolic blood pressure [DBP]: 153 ± 6 vs. 116 ± 4 mmHg and mean arterial pressure [MAP]: 174 ± 6 vs. 126 ± 5 mmHg; P < 0.001 for each comparisons) and AB_HF_ (SBP: 214 ± 6 vs. 146 ± 6 mmHg, DBP: 155 ± 3 vs. 116 ± 4 mmHg and MAP: 174 ± 4 vs. 126 ± 5 mmHg; P < 0.001 for each comparison) groups compared to the sham group. Nevertheless, SBP, DBP and MAP did not differ between the AB_LVH_ and AB_HF_ groups (P = 0.713 for SBP, P = 0.791 for DBP and P = 0.988 for MAP).

**Figure 1:**
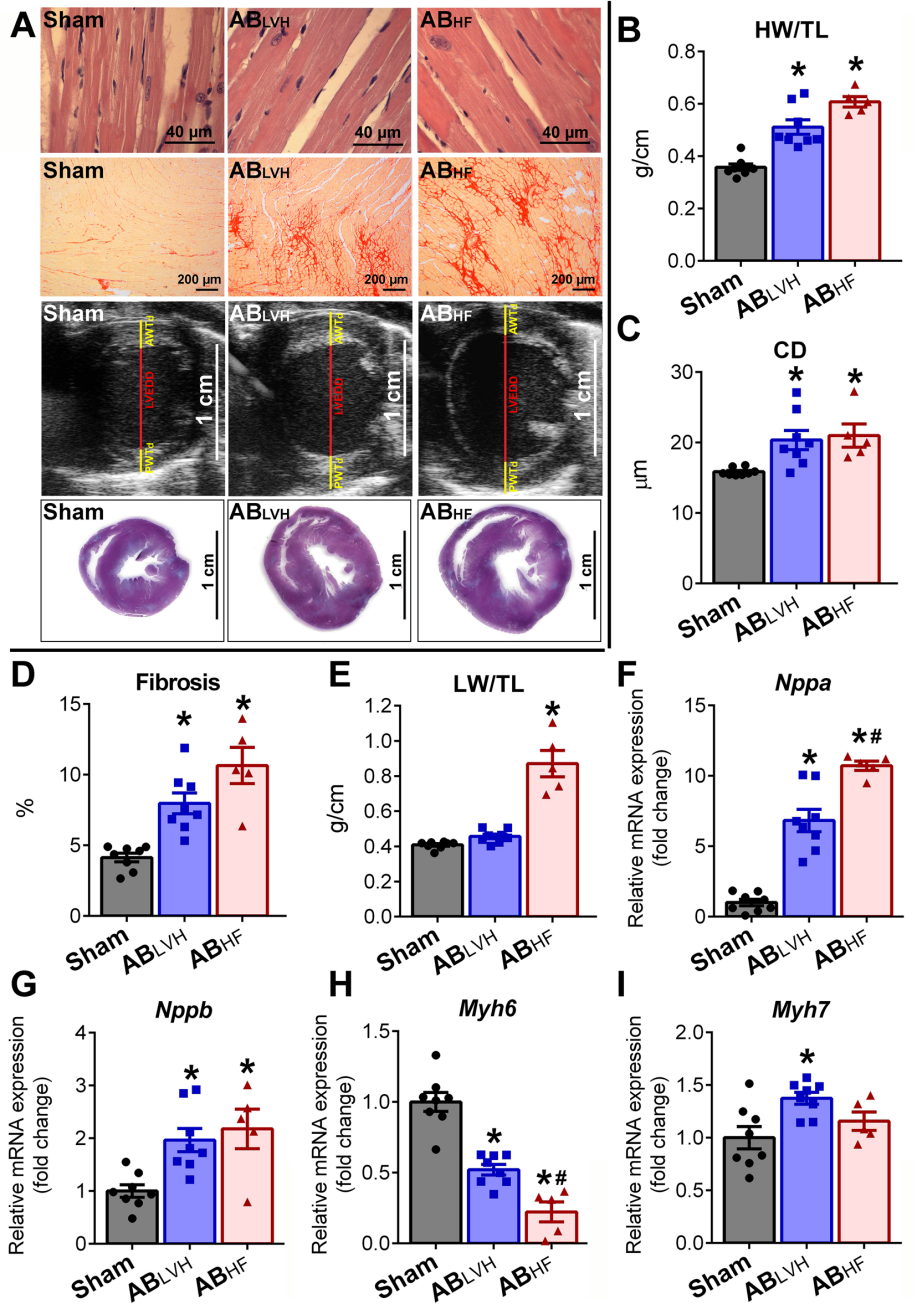
Model characterization. (**A**) Representative photomicrographs of hematoxylin and eosin (magnification 200 ×, scale bar: 40 µm) and picrosirius red staining (magnification 50 ×, scale bar: 200 µm), parasternal short axis echocardiographic recordings at the midpapillary muscle level and cross-sectional whole heart images are shown demonstrating myocardial hypertrophy and intensified fibrosis in the aortic-banded (AB) groups. (**B**) Heart weight-to-tibial length (HW/TL), (**C**) cardiomyocyte diameter (CD), (**D**) interstitial fibrosis, (**F**) atrial (*Nppa*) and (**G**) B-type natriuretic peptide (*Nppb*) mRNA expression were increased, while (**H**) myosin heavy chain alpha (*Myh6*) mRNA expression was decreased in the aortic-banded (AB) groups with systolic heart failure (AB_HF_) and also without systolic heart failure (AB_LVH_) compared to the sham group. The decrease in *Myh6* and the increase in *Nppa* was more severe in the AB_HF_ compared to the AB_LVH_ group. In contrast, (**I**) *Myh7* showed elevated levels only in the AB_LVH_ group when compared to its control group. Lung weight-to-tibial length (LW/TL) (**E**) increased only in the AB_HF_ group, indicating severe pulmonary congestion. Sample numbers were the following: Sham: n = 8, AB_LVH_: n = 8, AB_HF_: n = 5. Depending on the distribution of the datasets, one-way analysis of variance (ANOVA) followed by Tukey’s post hoc test or Kruskal–Wallis test followed by Dunn’s post hoc test was carried out. *P < 0.05 vs. Sham. ^#^P < 0.05 vs. AB_LVH_.

Furthermore, echocardiography revealed increased anterior wall thickness in diastole (AWT_d_), posterior wall thickness in diastole (PWT_d_) and LV mass in the AB_LVH_ and the AB_HF_ groups at week 6 and 12, when compared to sham group (Table 1). Heart weight-to-tibial length (HW/TL) (Fig. 1A), cardiomyocyte diameter (CD) (Fig. 1B), interstitial myocardial fibrosis (Fig. 1C) as well as the mRNA levels of natriuretic peptides (natriuretic peptide type A [*Nppa*] and natriuretic peptide type B [*Nppb*]) (Fig. 1E,F) also showed significantly increased values in AB_LVH_ and AB_HF_, confirming the development of pathological LVH in both groups. In addition, the robust decrease in α-myosin heavy chain (*Myh6*) mRNA expression (Fig. 1G) coupled with slight changes in β-myosin heavy chain (*Myh7*) transcription (Fig. 1H) led to an increased *Myh7/Myh6* ratio on the mRNA level in both the AB_LVH_ (Sham vs. AB_LVH_, P = 0.027) and in the AB_HF_ (Sham vs. AB_HF_ P = 0.002) groups.

**Table 1:** Echocardiographic follow-up.

	Week 6			Week 12		
	*Sham* (n = 8)	*AB*_*LVH*_ (n = 8)	*AB*_*HF*_ (n = 5)	*Sham* (n = 8)	*AB*_*LVH*_ (n = 8)	*AB*_*HF*_ (n = 5)
Body weight, g	460 ± 13	480 ± 9	450 ± 15	549 ± 14	576 ± 12	543 ± 23
Heart rate, beats/min	360 ± 9	349 ± 19	365 ± 16	356 ± 14	353 ± 8	340 ± 13
LV AWT_d_, mm	1.92 ± 0.07	2.55 ± 0.10*	2.69 ± 0.11*	1.99 ± 0.06	2.65 ± 0.05*	2.78 ± 0.06*
LV AWT_s_, mm	3.44 ± 0.08	4.50 ± 0.21*	3.84 ± 0.12^#^	3.57 ± 0.11	4.25 ± 0.12*	3.92 ± 0.09
LVEDD, mm	8.05 ± 0.16	8.56 ± 0.14	8.71 ± 0.27	8.32 ± 0.13	8.87 ± 0.15	9.93 ± 0.46*^,#^
LVESD, mm	4.68 ± 0.19	4.97 ± 0.20	6.14 ± 0.45*^,#^	4.83 ± 0.12	5.53 ± 0.26	7.59 ± 0.60*^,#^
LV PWT_d_, mm	2.00 ± 0.08	2.73 ± 0.09*	2.60 ± 0.12*	2.00 ± 0.07	2.50 ± 0.05*	2.46 ± 0.11*
LV PWT_s_, mm	3.24 ± 0.08	4.24 ± 0.10*	3.53 ± 0.21^#^	3.19 ± 0.10	4.13 ± 0.06*	3.26 ± 0.17^#^
LV mass, mg	1.25 ± 0.03	2.11 ± 0.07*	2.15 ± 0.10*	1.34 ± 0.04	2.15 ± 0.07*	2.65 ± 0.15*^,#^
LVEDV, µl	460 ± 14	518 ± 27	551 ± 48	511 ± 25	572 ± 25	734 ± 80*^,#^
LVESV, µl	202 ± 14	243 ± 21	369 ± 52*^,#^	211 ± 10	307 ± 21	605 ± 69*^,#^
SV, µl	258 ± 12	275 ± 26	182 ± 16^#^	300 ± 19	265 ± 15	130 ± 20*^,#^
CO, ml/min	97.9 ± 13.0	93.2 ± 5.7	65.9 ± 4.9	106.8 ± 7.9	93.8 ± 6.0	43.1 ± 5.8*^,#^
Ejection fraction, %	56 ± 2	53 ± 3	34 ± 5*^,#^	59 ± 2	47 ± 2*	18 ± 3*^,#^

LV left ventricular, *AWT*_*d*_ anterior wall thickness in diastole, *AWT*_*s*_ anterior wall thickness in systole, *LVEDD* LV end-diastolic diameter, *LVESD* LV end-systolic diameter, *PWT*_*d*_ LV posterior wall thickness in diastole, *PWT*_*s*_ LV posterior wall thickness in systole, *LVEDV* LV end-diastolic volume, *LVESV* LV end-systolic volume, *SV* stroke volume, *CO* cardiac output. The sample numbers are indicated in the table. Depending on the distribution of the datasets, one-way analysis of variance (ANOVA) followed by Tukey’s post hoc test or Kruskal–Wallis test followed by Dunn’s post hoc test was carried out. *P < 0.05 vs. Sham. ^#^P < 0.05 vs. AB_LVH_.

Despite the fact that pathological LVH developed in both the AB_LVH_ and AB_HF_ groups, chamber dilatation along with progressive decompensation of LV systolic function only occurred in the AB_HF_ and not in the AB_LVH_ group. Accordingly, while in the AB_LVH_ group systolic function was found to be only slightly deteriorated at week 12, ejection fraction (EF), stroke volume (SV) and cardiac output (CO) were massively reduced in the AB_HF_ group (Table 1). Similarly, LV end-diastolic volume (LVEDV) and LV end-diastolic diameter (LVEDD) were elevated in the AB_HF,_ but not in the AB_LVH_ group (Table 1). The manifestation of congestive HF in the AB_HF_ group was also reinforced by an elevated lung weight-to-tibial length (LW/TL) ratio (Fig. 1D).

### miRNA screening

**Figure 2:**
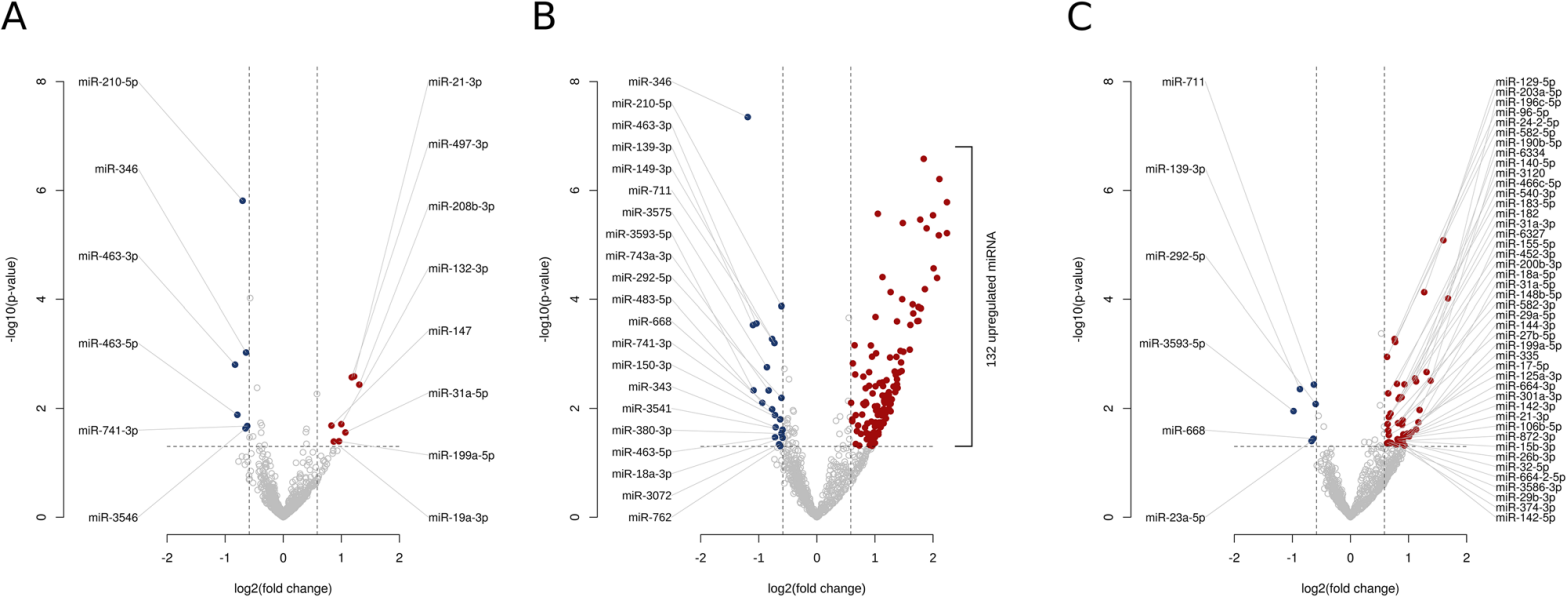
miRNA screening. Volcano plots presenting altered miRNA expression profiles of the different conditions. In the comparison of (**A**) aortic-banded (AB) groups without systolic heart failure (AB_LVH_) versus sham group, (**B**) AB groups with systolic heart failure (AB_HF_) versus sham group and (**C**) AB_HF_ group versus AB_LVH_ group 14, 153 and 50 miRNAs showed significant differential expression, respectively. Up- (red) and downregulated (blue) miRNAs that meet the specified significance threshold conditions (P-value < 0.05 and fold change > 1.5 or < 1/1.5) are listed (for the complete list of upregulated miRNAs in AB_HF_ group versus sham group see the Supplementary files).

miRNA sequencing identified 14 differentially expressed miRNAs in the AB_LVH_ group (Fig. 2A) and 153 in the AB_HF_ group (Fig. 2B) when compared to the Sham group (for the detailed lists please see Supplementary Tables 1 and 2). Importantly, 50 miRNAs showed different expression (*upregulated miRNAs*: rno-miR-106b-5p, 125a-3p, 129-5p, 140-5p, 142-3p, 142-5p, 144-3p, 148b-5p, 155-5p, 15b-3p, 17-5p, 182, 183-5p, 18a-5p, 190b-5p, 196c-5p, 199a-5p, 200b-3p, 203a-5p, 24-2-5p, 26b-3p, 27b-5p, 29a-5p, 29b-3p, 301a-3p, 3120, 31a-3p, 31a-5p, 32-5p, 335, 3586-3p, 374-3p, 452-3p, 466c-5p, 540-3p, 582-3p, 582-5p, 6327, 6334, 664-2-5p, 664-3p, 872-3p, 96-5p and *downregulated miRNAs*: rno-miR-139-3p, 23a-5p, 292-5p, 3593-5p, 668, 711) in the AB_HF_ compared to the AB_LVH_ group (Supplementary Table 3 and Fig. 2C). Considering that in the present study our aim was to uncover novel molecular targets which might contribute to the development of systolic HF in case of PO-evoked LVH, we focused on the comparison of AB_HF_ vs. AB_LVH_.

### Target prediction

**Figure 3:**
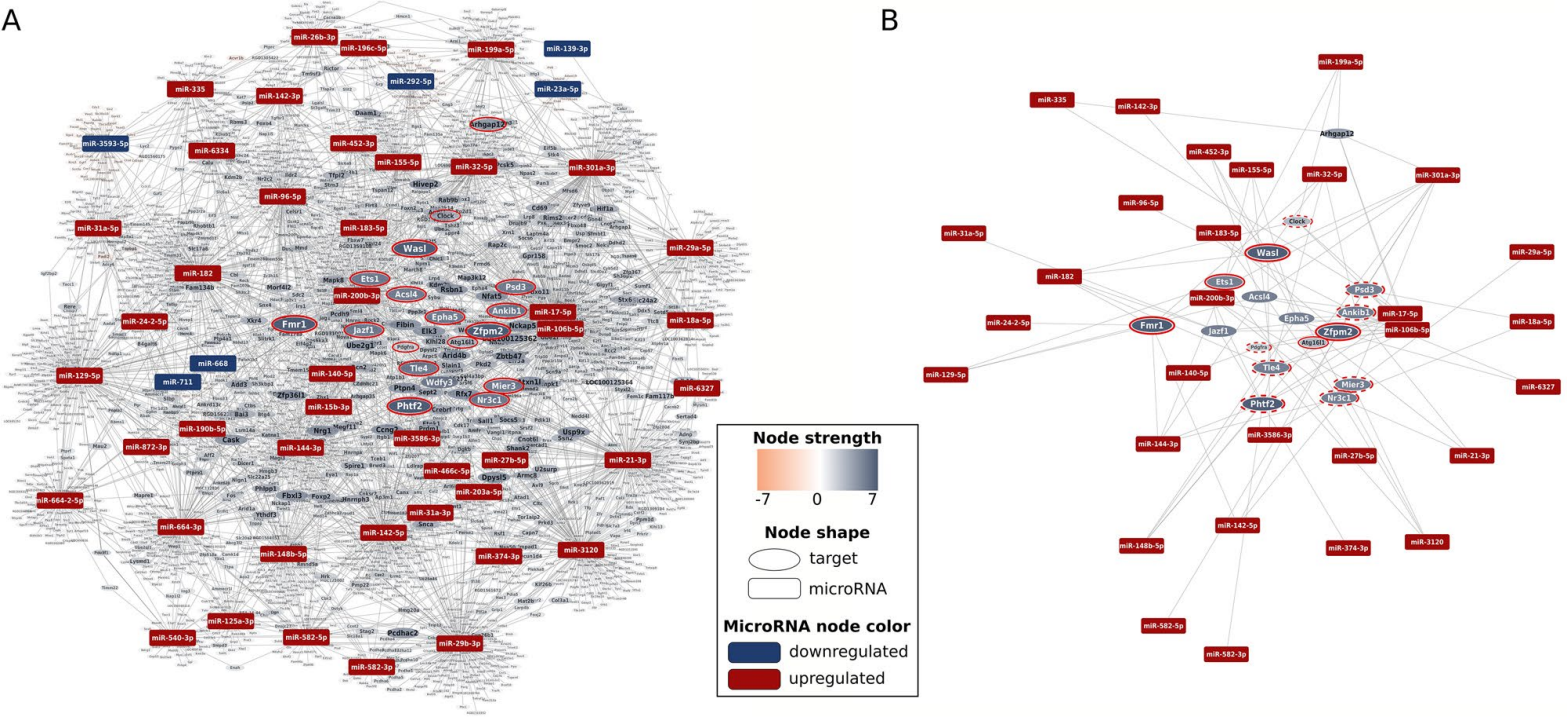
Target prediction. MiRNA-target interaction network presenting genes predicted to be involved in the maladaptive transition from left ventricular (LV) myocardial hypertrophy (LVH) into heart failure (HF). Up- and downregulated miRNA nodes are indicated with uniformly sized red and blue rectangles, respectively. Orange and blue ovals represent up- and downregulated target gene nodes, respectively. Node size and color intensity of target gene nodes increases according to the absolute node strength values. (**A**) Whole interaction network with red circles indicating genes selected to be validated on mRNA level. (**B**) Regulatory subnetwork of targets selected for validation. Targets with significant and non-significant expression change in the predicted direction are highlighted by continuous and dotted red circles, respectively. Expression change of target gene nodes without marking could not be observed.

Based on the results of miRNA transcriptomics, network theoretical miRNA-target analysis predicted 3007 genes (Fig. 3), the expression of which might have been differentially regulated by miRNAs in the AB_HF_ group compared to the AB_LVH_ group (for the complete list of genes please see Supplementary Table 4).

### Target validation

**Figure 4:**
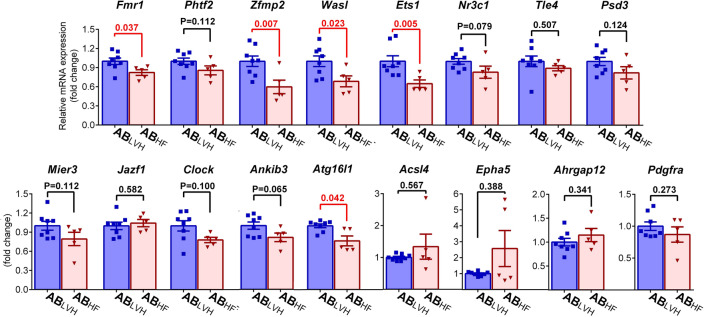
Target validation. Out of the tested 17 targets, five genes (*Fmr1*: FMRP translational regulator 1/fragile X messenger ribonucleoprotein 1, *Zfmp2*: zinc finger protein, multitype 2, *Wasl*: WASP like actin nucleation promoting factor, *Ets1*: ETS proto-oncogene 1 and *Atg16l1*: autophagy related 16-like 1) showed reduced mRNA expression in the AB_HF_ (aortic banded group with systolic heart failure) compared to the AB_LVH_ (aortic banded group without systolic heart failure) group. Furthermore, a tendency towards decreased values without reaching the level of significance was observed in case of *Phtf2* (putative homeodomain transcription factor 2), *Nr3c1* (nuclear receptor subfamily 3, group C, member 1). *Mier3* (MIER family member 3), *Clock* (clock circadian regulator) and *Ankib3* (ankyrin repeat and IBR domain-containing protein 1). No changes could be detected in mRNA expression between the two study groups in case of *Acsl4* (acyl-CoA synthetase long-chain family member 4), *Tle4* (TLE family member 4, transcriptional corepressor), *Wdfy3* (WD repeat and FYVE domain containing 3), *Psd3* (pleckstrin And Sec7 Domain Containing 3), *Jazf1* (JAZF zinc finger 1) and *Epha5* (EPH receptor A5). Sample numbers were n = 8 in the AB_LVH_ and n = 5 in the AB_HF_ groups. Depending on the distribution of the datasets, two sample Student’s *t* test or Mann–Whitney tests were carried out. Data normalized to the AB_LVH_ are depicted. *P < 0.05.

For validation purposes, target genes with the highest absolute node strength values were selected. 4 genes (*F*MRP translational regulator 1/fragile X messenger ribonucleoprotein 1 [*Fmr1*]; putative homeodomain transcription factor 2 [*Phtf2*]; zinc finger protein, multitype 2 [f*Zfpm2*] and WASP like actin nucleation promoting factor [*Wasl*]) showed a node strength value of 7. Furthermore, expression of another ten genes (acyl-CoA synthetase long-chain family member 4 [*Acsl4*], ETS proto-oncogene 1[*Ets1*], nuclear receptor subfamily 3, group C, member 1 [*Nr3c1*], TLE family member 4, transcriptional corepressor [*Tle4*], WD repeat and FYVE domain containing 3 [*Wdfy3*], pleckstrin And Sec7 Domain Containing 3 [*Psd3*], MIER family member 3 [*Mier3*], ankyrin repeat and IBR domain-containing protein 1 [*Ankib1*], JAZF zinc finger 1 [*Jazf1*], EPH receptor A5 [*Epha5*]) were found to be unidirectionally modulated by 6 miRNAs. The mRNA expression level of all these genes (except for *Wdfy3*, the expression of which could not be measured due to methodological reasons) coupled with four randomly selected genes, with predicted strong regulation (autophagy related 16-like 1 [*Atg16l1*]*,* Rho GTPase Activating Protein 12 [*Arhgrap12*]*,* clock circadian regulator [*Clock*]*,* Platelet Derived Growth Factor Receptor Alpha [*Pdgfra*]) were measured. Out of the tested 17 genes, *Fmr1*, *Zfmp2*, *Wasl*, *Ets1* and *Atg16l1* showed reduced mRNA expression (Fig. 4).

## Discussion

The present paper confirmed that LV miRNA expression is characteristically different in PO-induced LVH with systolic HF compared to PO-evoked LVH without systolic HF. Furthermore, bioinformatic analysis revealed that the altered miRNA profile in PO-evoked systolic HF might have led to the dysregulation of several genes. Importantly, out of the most promising predicted targets, *Fmr1*, *Zfpm2*, *Wasl*, *Ets1* and *Atg16l1* showed reduced expression on the mRNA level in PO-induced LVH with systolic HF.

The maladaptation of the LV in response to pure chronic PO (without an ischemic insult or severe valvular regurgitation leading to concomitant LV volume overload) characteristically involves the development of pathological LVH along with cardiomyocyte hypertrophy, interstitial fibrosis, impaired diastolic function but preserved or only mildly reduced systolic function^2,15,16^. On the other hand, advanced systolic HF with marked chamber dilatation manifests in a smaller portion of patients/experimental animals^3,17^. Data is scarce regarding the reasons and pathological mechanism for the different remodeling processes in case of sustained LV PO. Experimental studies indicate that the severity of PO is a major determinant of systolic functional impairment. Indeed, a higher degree of PO was found to predispose for contractility deterioration and LV dilatation in small animal models^18^. Furthermore, Richards et al. demonstrated that systolic dysfunction and elevated HF markers (plasma BNP level as well as LV ANP and BNP mRNA expression) were only present in mice with transverse aortic constriction (TAC) when the severity of PO exceeded a distinct level^4^. Based on these results, it could be hypothesized that if the extent of the pathological stimulus of PO reaches a critical “threshold” level, a more fulminant disease progression takes place. However, this notion is further complicated by the fact that only a portion of the animals developed decompensated systolic HF in well-controlled preclinical studies (where the degree of PO was highly standardized)^3,5^. In addition, in patients with AS, the degree of pathological LVH was only weakly related to the severity of valve obstruction^16^, but its extent was strongly associated with systolic dysfunction and HF development^19^. Therefore, it could be more precisely stated that a more severe PO increases the risk for systolic HF on the basis of individual susceptibility.

In our study the abdominal AB rat model was utilized, in which the LV was subjected to a moderately severe PO. In recent years, our research group has provided a detailed characterization of the natural progression of PO-evoked LVH in this particular model^20,21^. During these consecutive studies we have observed that myocardial hypertrophy along with typical pathological gene expressional changes (reactivation of the fetal gene program), interstitial fibrosis and impaired diastolic function (predominantly with prolonged active relaxation) develops relatively early after AB induction (at week 6)^21^. At this early stage, global systolic function is characteristically preserved. However, a moderate but progressive decline in LV contractility could be noted at later stages (after week 12)^21^. In parallel to our prior findings, the majority of the AB animals (43/49, 87.8%) demonstrated preserved systolic LV function at week 6 and slightly decreased EF at week 12 (Table 1). Furthermore, as also found earlier, development of LVH (as indicated by increased HW/TL, LV mass, AWT_d_, PWT_d_ and CD) in the AB_LVH_ was associated with reactivated fetal genes (elevated nppa, nppb and myh7 expression and decreased myh6 expression) and interstitial collagen accumulation (Fig. 1). In contrast to this classical course of PO-induced LVH, in a small subpopulation of the AB animals (6/49, 12.2%), rapid progression into decompensated systolic HF with massive pulmonary congestion (as reflected by the increased wet LW/TL ratio [Fig. 1D]) was noted. Analysis of the echocardiographic data revealed that systolic function was severely impaired already at week 6 in these rats, indicating that the remodeling processes were fundamentally separated from each other at a relatively early time point. Importantly, wall thickening did not differ between the AB_HF_ and AB_LVH_ groups. Similarly, no differences in pathological mechanism could be observed either in interstitial collagen accumulation, natriuretic peptide expression (indicating a comparable level of ventricular wall stress) or in MHC isoform switch (although myh6 showed more reduction in AB_HF_ and myh7 demonstrated more increase in AB_LVH_, the overall shift in the ratio did not differ [P = 0.242] between the groups [not depicted]). It is also worth highlighting that the extent of afterload increment (as reflected by SBP, DBP and MAP) was similar in the AB groups, indicating that the observed phenotypic differences could not simply originate from differences in hemodynamic loading conditions.

To better understand the underlying molecular mechanisms for the observed phenotypic differences in the AB_LVH_ and AB_HF_ groups, LV miRNA transcriptomic profiling followed by network theoretical miRNA-target analysis was carried out. Our decision to use a miRNA-based, unbiased target prediction approach to identify genes, the altered expression of which could have contributed to the decompensation of systolic function in the AB_HF_ group was rationalized by two reasons. First, a great body of scientific evidence supports the fact that LV miRNA expression is substantially dysregulated in PO-evoked pathological LVH^13,14^. Hence, marked changes in the LV miRNAome could have been anticipated to occur in our model as well. Secondly, the effectiveness of miRNA interactome analysis in the identification of important, post-transcriptionally regulated genes in different cardiovascular pathologies have been well established by earlier studies^8–12^.

The NGS-derived miRNA expressional data was compared between the study groups in all the possible scenarios (AB_HF_ vs. Sham, AB_LVH_ vs. Sham and AB_HF_ vs. AB_LVH_; see also Fig. 2). Considering the fact that the direct comparison of PO-induced LVH with and without systolic HF carries the potential to filter out those pathological molecular alterations that might have specifically contributed to the deterioration of systolic function and chamber dilatation (and not to the development of LVH), the comparison of AB_HF_ to AB_LVH_ was chosen for further evaluation. This comparison revealed 44 upregulated and 6 downregulated miRNAs in the AB_HF_ compared to the AB_LVH_ group (Fig. 2C). Based on these results, our network analysis method predicted more than 3000 differentially regulated genes (Fig. 3). Given the big number of targets, only 17 genes with high node strength values were analyzed on the mRNA level. This validation process confirmed reduced expression of the following five genes: *Fmr1*, *Zfpm2*, *Wasl*, *Ets1* and *Atg16l1* in the AB_HF_ group compared to the AB_LVH_ group (Fig. 4), suggesting a potential role of these genes in the development of systolic HF.

Among these target genes, *Fmr1* encodes a protein called fragile X messenger ribonucleoprotein 1^22^, which is mainly required for physiological cognitive development. Mutation of the *Fmr1* gene (classically due to trinucleotide repeat expansion) leads to fragile X syndrome (FXS)^23^. Although cardiovascular abnormalities are not uncommon in FXS^24^, the exact role of *Fmr1* in the heart is not completely understood. A recent study suggested that *Fmr1* might have some cardioprotective properties, since its overexpression alleviated oxidative stress and apoptosis in an in vitro model of lipopolysaccharide-induced myocardial injury^25^. Furthermore, previous experiments confirmed that genetic silencing of *Fmr1* (by knocking the gene out [KO]) was associated with increased iron content in the adult myocardium^26^, and premature closure of the mitochondrial permeability transition pore in the developing heart^27^. Nevertheless, the significance of these observations on the cardiovascular system warrants further investigations, as newborn *Fmr1* KO mice do not exhibit LV dysfunction^27^.

Another interesting target, the reduced expression of which was successfully validated in our study, was the multizinc finger protein *Zfpm2* gene (also known as the friend of GATA [FOG]-2). Importantly, the essential role of ZFPM2/FOG-2 in the normal morphogenesis of the heart and in the effective vascularization of the myocardium has been well documented^28^. Correspondingly, genetic deletion of *Zfpm2* during embryonic development results in severe defects in cardiac structure and coronary artery formation^29^. Of particular significance, cardiomyocyte-specific loss of the *Zfpm2* gene product in adult mice did not lead to anatomical disturbances, but resulted in severely depressed ventricular function and premature mortality^29^. Taking these data together, it is tempting to hypothesize that reduction in *Zfpm2* expression might have inhibited the normal vascularization of the hypertrophied myocardium, leading to tissue hypoxia and consequently to reduced LV contractility in the AB_HF_ group.

The protein coded by *Wasl* associates with a variety of signaling molecules that regulate the actin cytoskeleton system. Genetic mutation of *Wasl* results in Wiskott-Aldrich syndrome (WAS), which is characterized by immunodeficiency, thrombocytopenia and atopic dermatitis^30^. The cardiovascular system is characteristically not affected in WAS. Unfortunately, previous publications did not provide a detailed characterization of cardiac function and structure in *Wasl* KO mice models. Hence, further experimentation is required to uncover the potential role of *Wasl* in HF.

*Ets1* encodes a transcriptional factor that plays a pivotal role in cardiac lineage commitment from a pluripotent stage during early embryonic development^31^. Its defect has been also associated with LV non-compaction^32^. Furthermore, ETS1 is required for the proper development of the coronary artery system by regulating epithelial-mesenchymal transformation^33^. This later finding suggests that (besides *Zfpm2*) the reduced expression of *Ets1* could have also resulted in inappropriate vascularization of the pathologically remodeled LV in the AB_HF_ group.

Finally, the protein product of *Atg16l1* connects to the ATG5–ATG12 conjugate to form the ATG5–ATG12–ATG16L1 complex, which is essential for proper autophagosome formation^34^. Of great significance, the deleterious effect of autophagy inhibition in PO-evoked systolic HF was elegantly demonstrated in a prior publication, in which *Atg5* deficient mice exhibited LV dilatation and severe systolic dysfunction early after PO induction^35^. Although, the same experiment was not carried out with *Atg16l1* KO mice, one might speculate that the reduced expression of *Atg16l1* would also result in impaired function of the ATG5–ATG12–ATG16L1 complex and hence it would also negatively affect the remodeling process in response to sustained PO.

### Study limitations

Due to the low appearance rate of the AB_HF_ phenotype (~ 10%), relatively small sample sizes could be utilized in the present study. To avoid imbalances in sample numbers among the study groups, 8 out of 43 AB animals demonstrating conventional disease progression were randomly selected to form the AB_LVH_. Therefore, molecular analysis was only performed in the minority of AB_LVH_ rats. Furthermore, it is also worth mentioning that the presence of air-filled intestines placed around the operated abdominal aorta did not allow us to directly assess the severity of aortic constriction by non-invasive Doppler ultrasound. Hence, the extent of PO could be only determined via invasive hemodynamic measurement at the end of the study protocol. In addition, the interpretation of results is limited to young male rats. Therefore, further experimentation is required to investigate whether our observations could also be extended to female and older animals as well. Finally, it also has to be considered that the presence of numerous comorbidities (most importantly myocardial ischemia) and pharmacological HF therapies as well as the fundamental differences between the human pathophysiological states of sustained LV PO (long standing AS or arterial hypertension) and the AB rat model significantly limit the direct translation of our molecular findings into the clinical arena. Indeed, further validation of our results on human myocardial samples is necessary.

Besides the above-mentioned model-associated concerns, our molecular measurements also contain limitations. Of particular significance, the mRNA levels of the predicted target genes were measured in order to directly detect the miRNA-driven post-transcriptional regulations. Nevertheless, further investigation is needed to assess whether the observed gene expressional alterations indeed manifest in altered protein levels. Furthermore, since the global myocardial miRNA transcriptomic profiles were only assessed to provide input for the network theoretical prediction of the most relevant targets, our work did not focus either on individual miRNA level measurements by qPCR or on functional interaction analysis between distinct miRNAs and target genes.

## Conclusion

LV miRNA expression is characteristically different in PO-evoked LVH with and without systolic HF in a rat model of abdominal AB. Furthermore, the changes of myocardial miRNA expression are associated with the dysregulation of a great number of genes in the AB_HF_ compared to the AB_LVH_ groups. Out of the most prominent targets, reduced expression was confirmed in the following 5 genes: *Fmr1*, *Zfpm2*, *Wasl*, *Ets1* and *Atg16l1*.

## Methods

### Animals

The investigation conformed to the EU Directive 2010/63/EU and the Guide for the Care and Use of Laboratory Animals used by the US National Institutes of Health (NIH Publication No. 85-23, revised 1996) and all of the methods were performed according to these proper guidelines and regulations. The experiments were approved by the ethics committee of the Regional Council of Karlsruhe for Animal Experimentation (G-94/15). Furthermore, the study is also interpreted in accordance with the ARRIVE (Animals in Research: Reporting in Vivo Experiments) guidelines^36^. Male Sprague–Dawley rats (n = 63; 5–6 weeks-old; 160–180 g; Janvier Labs, Saint Berthevin, France) were kept under standard conditions (22 ± 2 °C with 12 h light/dark cycles) and were allowed access to laboratory rat diet and water ad libitum.

### Abdominal aortic banding

After a one-week-long acclimatization period, abdominal AB (n = 55) or sham operation (n = 8) was performed. In brief, under isoflurane anesthesia a midline laparotomy was carried out. Then, the intestinal tract was gently placed aside and the peritoneal layer was dissected in order to gain access to the retroperitoneal space. The abdominal aorta between the right renal artery and the superior mesenteric artery was carefully cleaned from the surrounding connective tissue. After isolation, a blunted 22-gauge needle (with the external diameter of 0.72 mm) was placed on the anterior surface of the abdominal aorta and a surgical thread was looped below the vessel and the needle. In rats undergoing AB surgery, the surgical thread was then subsequently tightened and the needle was removed, inducing a constriction of the size of the applied needle. After AB was completed, the intestines were placed back to the abdominal cavity and the abdominal muscle layer was sutured in single interrupted fashion. Finally, the skin wound was closed by applying surgical clips. Following surgery, analgesia was provided by subcutaneously administered buprenorphine in the dose of 0.05 mg/kg BW. Sham-operated animals were subjected to the same surgical procedure, except the aortic constriction.

### Experimental groups

From the 55 rats undergoing AB, 5 died in the subacute phase (within the first 72 h) and one during the follow-up period. Hence, the final sample size in the AB group was 49 at the end of the experimental period. From these 49 AB rats, progressive transition from pathological LVH (AB_LVH_) into systolic HF occurred in 6 rats (12.2%) (termed as AB_HF_ group) at week 12. An ejection fraction of less than 30% measured by echocardiography at week 12 was determined as an inclusion criterion for the AB_HF_ (based on the current definition of advanced systolic HF)^37^. Accordingly, the experimental groups were the following:

Sham group (n = 8): these rats underwent sham operation and a 12-week long follow-up period.

AB_LVH_ group (n = 8): these rats underwent AB operation and a 12-week long follow-up period and an EF of more than 30% was measured by echocardiography at the end of the experimental period. 43 rats met this criterion, however to match the sample size of this group to the other two experimental groups, only 8 rats were randomly selected for further analysis.

AB_HF_ group (n = 5): these rats underwent AB operation and a 12-week long follow-up period and an EF of less than 30% was measured by echocardiography at the end of the experimental period. In one of the AB_HF_ rats a sudden death occurred during the final echocardiographic measurement and therefore invasive blood pressure monitoring as well as tissue collection for histological and molecular analysis could not be carried out. Therefore, this animal was excluded from further analysis.

### Echocardiography

Echocardiography was carried out using the Vevo^®^ 2100 imaging system (FujiFilm VisualSonics Inc., Toronto, Ontario, Canada) equipped with a 21-MHz linear probe according the previously published protocol^38^. Repetitive measurements were performed at 6 and 12 weeks after AB/Sham operation. In brief, rats were anesthetized with 5% isoflurane in a chamber. After the induction of anesthesia, rats were placed on an automatic heating pad in a supine position with core temperature maintained at 37 °C. The thorax of the animals was shaved to obtain an optimal acoustic window. Anesthesia was maintained by inhalation of 1–1.5% isoflurane gas in 100% O_2_. Images in two-dimensional parasternal long-axis (PLAX) and short-axis views (PSAX) as well as M-mode recordings at the midpapillary level were taken. From PLAX acoustic window, LV longitudinal diameters (from LV apex to the aortic root) and transverse diameters (at the mid-papillary level) were measured at LVESD and LVEDD. On M-mode recordings (also at the mid-papillary level), AWT and PWT in diastole (d) and systole (s) were determined. LVESV and LVEDV were determined by the biplane ellipsoid model. SV and CO were calculated based on the following equations: SV = LVEDV-LVESV, CO = SV*heart rate (HR). LV mass was assessed according to the Deveroux formula.

### Invasive hemodynamics

After completion of the echocardiographic protocol, in vivo hemodynamic measurements were performed. Briefly, rats were tracheotomized and intubated to facilitate breathing. Anesthesia was supported by mechanical ventilation with 1–1.5% isoflurane gas in 100% O_2_. A 2-Fr pressure-conductance microcatheter (SPR-838; Millar Instruments, Houston, TX) was then inserted into the right carotid artery and advanced into the ascending aorta. After stabilization, aortic pressure curves were recorded to measure and calculate SBP, DBP and MAP.

### Post-mortem organ measurements

Following the invasive hemodynamic measurement, median laparotomy was performed and the abdominal aorta was isolated and cannulated. The blood of the animals was collected via the inserted cannula. To remove the residual blood cells from myocardial tissue, retrograde perfusion with cold physiological saline was applied. After perfusion, the hearts and the lungs of the animals were removed from the thorax and their weights were quickly measured on a scale. This was followed by conservation of LV myocardial tissue. Accordingly, transverse segments (the middle third part) of the right and the LV were fixed in buffered paraformaldehyde solution (4%) and embedded in paraffin for histological analysis. Furthermore, the apex of the LV was cut into small pieces (40–50 mg) and subsequently snap frozen in liquid nitrogen for molecular measurements. After tissue conservation was completed, tibial length (TL) was measured and the ratios of HW/TL and LW/TL were calculated.

### Histology

Transverse, transmural, 5-µm thick slices of the ventricles were cut and placed on adhesive slides. These sections were stained with hematoxylin and eosin staining to determine CD as a cellular marker of myocardial hypertrophy^38^. In each sample, 100 longitudinally oriented cardiomyocytes from the LV were examined, and the diameters at transnuclear positions were defined with ImageJ software (National Institutes of Health, Bethesda, MD). The mean value of 100 measurements represented one sample.

The extent of interstitial myocardial fibrosis was assessed on picrosirius-stained sections, as also described earlier^39^. ImageJ software (National Institutes of Health, Bethesda, MD) was used to identify the picrosirius-red positive area. Three images (magnification 50 ×) were randomly taken from the free LV wall on each sections. After background subtraction, eye-controlled auto-threshold has been determined to detect positive areas. The collagen area (picrosirius red positive area-to-total area ratio) was determined on each image, and the mean value of three images represents each animal. The evaluation of the histological sections was performed by an independent observer who was blinded to the experimental design.

### LV miRNA and mRNA expression analysis

#### Sample preparation

RNA isolation was performed from 50 mg of LV myocardial tissue using the RNeasy Plus Universal kit (Qiagen, Hilden, Germany) according to the manufacturer’s instructions paying special attention to appendix D in order to ensure purification of total RNA including small RNAs. Isolated RNA was subsequently eluted in 30 µl RNase-free water.

#### Quality control of RNA

RNA quality and concentration were determined and reviewed using a NanoDrop^®^ spectrophotometer (Thermo Scientific™, Waltham, MA, USA) to measure the concentration and optical density (OD) 260/280 values of the samples and an Agilent RNA TapeStation^®^ (Agilent Technologies, Santa Clara, USA) to check RNA quality via RINe number. RINe > 5 were accepted as input for library preparation. Quantification was performed via absorbance measurements using Qubit RNA broad range (BR) Assay Kit (Thermo Scientific™, Waltham, MA, USA).

#### Sequencing library

Library preparation was done using the QIAseq^®^ miRNA Library Kit (Qiagen, Hilden, Germany) following the manufacturer’s instructions. A total of 500 ng of RNA per sample was converted into miRNA next generation sequencing (NGS) libraries. Primers containing UMIs were used during reverse transcription. The resulting cDNA was amplified using PCR (13 cycles) with universal forward and sample index assigning reverse primers. The PCR product was then purified. Quality control of the library preparation was attained by capillary electrophoresis using the High Sensitivity Tape D1000 (Agilent Technologies, Santa Clara, USA). Based on the quality of the inserts and the concentration measurements, the libraries were pooled in equimolar ratios. The library pool(s) were quantified using qPCR and were then sequenced on a NextSeq^®^ Sequencing System (Illumina, San Diego, USA) according to the manufacturer instructions with 75 bp read length for read 1 and 8 bp for the index read (1 × 75, 1 × 8). Raw data was demultiplexed and FASTQ files for each pool were generated using the bcl2fastq Conversion Software (Illumina, San Diego, USA).

#### cDNA synthesis and qPCR

For cDNA synthesis the Qiagen RT^2^ First Strand Kit (cat. no. 330401) was used. This kit provides a rapid and convenient procedure for efficient first strand cDNA synthesis. The cDNA was mixed with an appropriate RT2 SYBR^®^ Green ROX Mastermix (cat. no. 330529). This mixture was aliquoted into the wells of the Custom RT^2^ Rat PCR Array (CLAR40805). PCR was performed on the ViiA 7 Real-Time PCR System (Thermo Scientific™, Waltham, MA, USA), and finally relative expression (for the following genes: *Acsl4, Arhgap12, Ankib1, Atg16l1, Clock, Ets, Epha5, Fmr1, Jazf1, Mier3, Myh6, Myh7, Nppa, Nppb, Nr3c1, Pdgfra, Phtf2, Psd3, Tle4, Wasl, Zfpm2*) normalized to housekeeping genes was determined using data from the real-time cycler and the 2^−ΔCT^ method. Regarding the qPCR measurements, one sample from the AB_HF_ group was excluded from data analysis due to technical issues.

### Network theoretical miRNA-target prediction

After selecting significantly differentially expressed miRNAs identified in different comparisons the network theoretical miRNAtarget™ (http://mirnatarget.com; Pharmahungary, Szeged, Hungary) software was used for miRNA-target prediction as in previous works^8–12,40^. miRNAtarget™ software integrates both experimentally validated (miRTarBase v7.0^41^) and predicted (miRDB v5.0 with score > 80.0^42^ and microRNA.org release of August 2010 with mirSVR score < − 1.2^43^) miRNA-target interaction databases to enable reliable predictions. In the predicted miRNA-target interaction networks nodes and edges represent miRNA and target entities and interactions between them, respectively. Weight of the edges originating from up- and downregulated miRNA nodes were defined to be + 1 and − 1, respectively. Node strength values are provided as the measure of the predicted expression change of the given target node, calculated as the sum of edge weights of the associated edges. Therefore, negative and positive node strength values indicate predicted up- and downregulation of the targets, respectively. miRNA-target interaction networks were visualized using Cytoscape software platform^44^ with the EntOptLayout plugin^45^.

### Statistics

The distribution of the datasets was tested by Kolgomorov–Smirnov normality test. All values are expressed as mean ± standard error of the mean.

An unpaired two-tailed Student’s *t*-test in case of normal distribution or Mann–Whitney *U* test in case of non-normal distribution was used to compare two independent groups.

To compare three independent groups, one-way analysis of variance (ANOVA) followed by Tukey’s post hoc test (in case of normal distribution) or Kruskal–Wallis test followed by Dunn’s post hoc test (in case of not normal distribution) were carried out.

A P-value of < 0.05 was used as the criterion of statistical significance. In addition, for declaring significant differential expressions a fold change threshold of 1.5 (fold change > 1.5 or < 1/1.5) was also considered.

### Supplementary Information

Supplementary Table 1.

Supplementary Table 2.

Supplementary Table 3.

Supplementary Table 4.

## Data Availability

The datasets from the RNA sequencing experiments are deposited and available at ArrayExpress (https://www.ebi.ac.uk/biostudies/arrayexpress) under the accession number of E-MTAB-12671.
